# Particle Filtering for Obstacle Tracking in UAS Sense and Avoid Applications

**DOI:** 10.1155/2014/280478

**Published:** 2014-07-01

**Authors:** Anna Elena Tirri, Giancarmine Fasano, Domenico Accardo, Antonio Moccia

**Affiliations:** University of Naples “Federico II”, I80125 Naples, Italy

## Abstract

Obstacle detection and tracking is a key function for UAS sense and avoid applications. In fact, obstacles in the flight path must be detected and tracked in an accurate and timely manner in order to execute a collision avoidance maneuver in case of collision threat. The most important parameter for the assessment of a collision risk is the Distance at Closest Point of Approach, that is, the predicted minimum distance between own aircraft and intruder for assigned current position and speed. Since assessed methodologies can cause some loss of accuracy due to nonlinearities, advanced filtering methodologies, such as particle filters, can provide more accurate estimates of the target state in case of nonlinear problems, thus improving system performance in terms of collision risk estimation. The paper focuses on algorithm development and performance evaluation for an obstacle tracking system based on a particle filter. The particle filter algorithm was tested in off-line simulations based on data gathered during flight tests. In particular, radar-based tracking was considered in order to evaluate the impact of particle filtering in a single sensor framework. The analysis shows some accuracy improvements in the estimation of Distance at Closest Point of Approach, thus reducing the delay in collision detection.

## 1. Introduction

Unmanned aircraft systems (UAS) must guarantee an equivalent level of safety compared to manned aircraft in order to be allowed flying in both controlled and uncontrolled airspace [1]. Thus, they must have the ability to “see and avoid” other aircraft and/or obstacles in the flight path, and they need to be equipped with an autonomous detect, sense, and avoid (DS&A) system [2, 3]. The primary functions of this system consist in detecting obstacles in the own trajectory, tracking the detected objects, and executing a collision avoidance maneuver if an intruder is closely approaching, thus becoming a collision threat. A Near Mid-Air Collision is determined when the distance between own aircraft and an intruder is less than 500 ft [3]; thus the prediction of the Distance at Closest Point of Approach (DCPA) is considered as a fundamental parameter for the estimation of a collision risk. In order to obtain an accurate DCPA estimate, an adequate sensor setup has to be chosen. In fact, the autonomous avoidance function can be performed installing onboard UAS cooperative and/or non-cooperative technologies [4–6].

In the framework of TECVOL project carried out by the Italian Aerospace Research Centre and the University of Naples “Federico II,” non-cooperative technology based on an integrated radar/electro-optical (EO) architecture has been considered as the optimal solution, since the project aimed at demonstrating the full autonomy of the system in case of absence/loss of data link between the aircraft [4, 7]. The prototype sensing setup has been installed onboard an optionally piloted very light aircraft named FLARE (flying laboratory for aeronautical research). The hardware/software architecture is comprised of radar and four electro-optical cameras and a central-level fusion algorithm based on an extended Kalman filter (EKF). An intensive flight test campaign has been performed taking into account several relative flight geometries including chasing and frontal encounters [8, 9] and considering a non-cooperative intruder of the same class of FLARE.

In general, while EKF is a well-assessed technique suitable for real time implementation, nonlinearities in obstacle dynamics and/or in the measurement equation can cause some loss of accuracy in obstacle tracking performance. On the other hand, innovative filtering techniques developed for nonlinear systems, such as particle filters, are expected to provide more accurate estimates of obstacle dynamics than EKF, thus improving the accuracy of the DCPA estimate and potentially reducing the delay in collision detection [10].

Thus, a research effort has been carried out in order to assess the impact of particle filtering approaches in the considered application.

In general, tracking problems comprise several applications such as ocean surveillance, submarine tracking, car positioning, video surveillance, and airborne target tracking. Different solutions and methods have been proposed and implemented [11–16].

One of the key components of a tracking system is the filtering and prediction block, where target motion is modeled using a state vector and a dynamic system, and sensor observations are related to the state vector by a proper measurement equation. Within this framework, most popular solutions are based on Kalman filtering [17–19] which can be demonstrated to be the theoretically optimal approach for linear systems corrupted by gaussian noise. In the latest years, particle filtering techniques have found increasing diffusion in problems involving nonlinear and non-Gaussian noise processes [20–23].

Particle filtering has been applied to various nonlinear problems, such as bearing-only tracking, tracking multitarget in clutter, and jump Markov linear system.

In [24], the tracking problem is examined in the context of autonomous bearing-only tracking of a single appearing/disappearing target in the presence of clutter measurements. The same problem is also analyzed in [21] where a comparison between EKF, a pseudo measurement tracking filter, and a PF is pointed out. The study aims at tracking a target from an airborne sensor at an approximately known altitude. The analysis shows the PF algorithm to be more robust than EKF. Karlsson and Gustafsson [25] make a comparison between the two filters for angle-only tracking in several applications such as air-to-air, air-to-sea, and sea-to-sea scenarios. In the study, experimental data are used to test the algorithm in the sea-to-sea case. The results show that the PF outperforms the EKF algorithm. Besides, PF has been also applied to solve problems of tracking multiple targets in clutter environment [26–28]. These works provide some implementation suggestions to improve the PF performance with extension to bootstrap filter.

This paper describes a customized particle filtering algorithm based on spherical coordinates and aimed at flying obstacle detection and tracking for non-cooperative sense and avoid. On the basis of application needs, the algorithm has been developed to understand PF potential especially in terms of reliability and latency in collision risk estimation. Thus, the predicted DCPA between aircraft has been considered as the key variable of the problem.

Besides the application field, main innovation aspects of the developed PF algorithm comprise the treatment and compensation of sensor latencies in view of real time software implementation, with the negative time measurement update problem dealt with keeping in memory a sliding window and using a Monte Carlo sampling approach and the direct particle-based prediction of minimum separation distance.

The paper is organized as follows. First, a brief description of problem geometry and hardware architecture of the system used in flight tests is provided. Then, the tracking algorithm is presented in detail. Finally, results obtained in off-line simulations based on flight data gathered during TECVOL test campaign are presented and discussed.

## 2. Obstacle Detection and Tracking for UAS Sense and Avoid

A DS&A system has to be endowed with an obstacle detection and tracking system that performs the “sense” function and a collision avoidance subsystem that performs a collision avoidance maneuver as soon as a collision risk is detected. In order to realize a suitable and reliable sense and avoid system, a series of requirements concerning the field of regard, range resolution, angular resolution, detection range, time to collision, and system data rate have to be satisfied in the case of non-cooperative technologies. In particular, flight regulations define the scanning volume for collision threat detection and the maximum relative speed between the two aircraft. Moreover, they prescribe that the own aircraft and the intruder must never be closer than 500 ft, that is, the so-called bubble distance [3].

**Figure 1:**
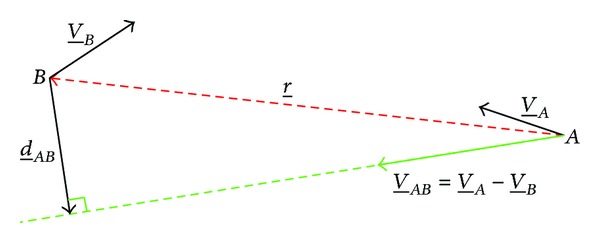
Definition of* minimum separation distance *vector ${\underline{d}}_{AB}$.

Considering the above-mentioned requirements, they are essentially relevant to the ability to estimate the Distance at Closest Point of Approach and the Time to Closest Point of Approach, often named “time to go” in the literature. The former parameter indicates the minimum distance that will be measured between two planes that keep their current velocity constant; it can be calculated from [4]:

(1) $$\begin{array}{c} {\underline{d}}_{AB}=\frac{\underline{r}\cdot {\underline{V}}_{AB}}{{||{\underline{V}}_{AB}||}^{2}}{\underline{V}}_{AB}-\underline{r}, \end{array}$$

where ${\underline{d}}_{AB}$ is the* minimum separation distance *(whose norm is the DCPA) [29], ${\underline{V}}_{AB}$ is the relative speed vector (${\underline{V}}_{A}-{\underline{V}}_{B}$) between the intruder (aircraft *A*) and the ownship (aircraft *B*), and $\underline{r}$ is the relative position between the aircraft (see Figure 1). If the intruder is modeled as a spherical object with radius *R*, it can be demonstrated [4] that assuming constant velocity vectors, ${\underline{V}}_{A}$ and ${\underline{V}}_{B}$, respectively, the two aircraft are headed for collision if and only if the following conditions are satisfied:

(2) $$\begin{array}{c} ||{\underline{d}}_{AB}||\leq R,\;\;\dot{r}<0. \end{array}$$

These values are a measure of the collision risk; if these conditions are satisfied, a Near Mid-Air Collision threat is considered.

It is worth noting that in order to obtain an accurate estimate of the DCPA, an adequate sensor setup has to be properly chosen. In general, this function can be performed installing onboard UAS cooperative and/or non-cooperative systems that include both active and passive sensors; the choice depends on size, speed, maneuverability, and missions for which the UAS will be used. In case of large UAS platforms and fully autonomous systems, integrated radar/electro-optical architecture seems to be a good option to increase the collision threat awareness. In fact, radars provide accurate range estimates and can operate in all-time all-weather conditions, while EO sensors offer the potential of accurate angular measurements at fast update rates; in addition, these systems do not rely on information from external sources to detect and track other aircraft, thus avoiding problems due to the loss of communication links.

As anticipated above, within TECVOL project a configuration with radar as main sensor and EO as auxiliary sensors has been chosen as the optimal solution in view of the application. In fact, this project aimed at the development and flight demonstration of the technologies needed to support high altitude long endurance UAS flight autonomy. The choice of a multisensor configuration allows a compensation of single sensor shortcomings by integrating information from heterogeneous sources. In the next section, for the sake of clarity the flight system architecture is briefly described.

## 3. Flight System Architecture

**Figure 2:**
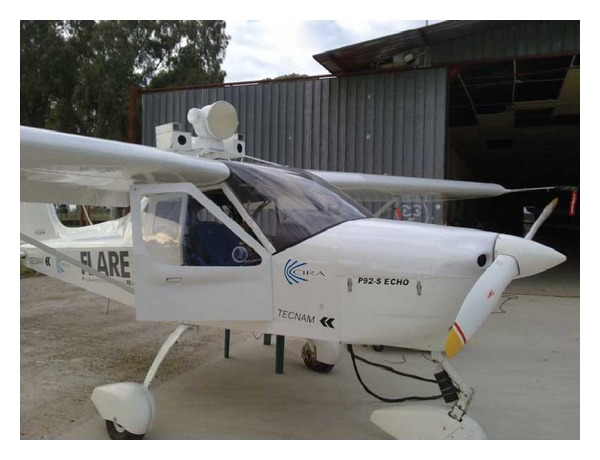
Sensing system onboard FLARE.

During the TECVOL flight test campaign, the prototype hardware/software sensing system installed onboard FLARE (Figure 2) was comprised of a Ka-band pulse radar and four electro-optical cameras (two visible cameras and two infrared cameras), a CPU devoted to image processing (IP-CPU), and a CPU devoted to real time tracking by sensor data fusion (RTT-CPU). This unit is connected to the onboard flight control computer that performs autonomous navigation and flight control, including autonomous collision avoidance maneuvers [4, 7, 30]. A hierarchical architecture in which the radar is the main sensor that must perform initial detection and tracking and the EO sensors are used as auxiliary information sources in order to increase accuracy and data rate has been considered. The choice of a hierarchical architecture is determined by different levels of sensor performance; in fact, the conventional EO sensors perform initial detection at closer ranges than radar. The standalone radar tracking estimates can be effective in reducing the computation time and false alarm rate of EO image processing by selecting properly defined search windows to apply the obstacle detection process.

**Figure 3:**
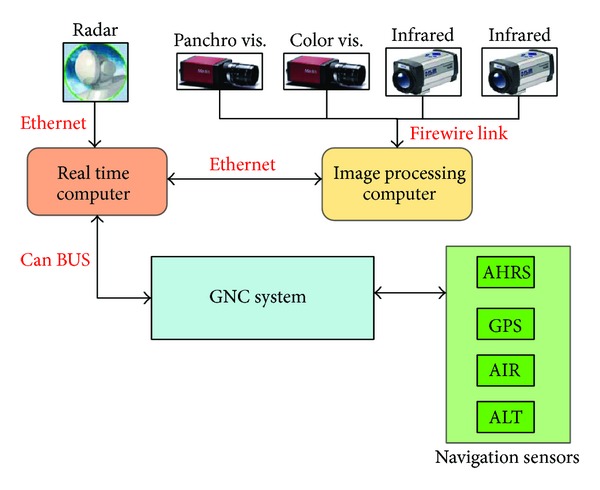
The onboard hardware architecture.

In Figure 3, the adopted hardware system is depicted. For more details on the hardware and logical architectures adopted within TECVOL project, the reader is referred to [4, 7].

The tracking system can operate in two different modes:

1. standalone radar tracking mode that performs current estimates of obstacle relative position and velocity on the basis of measurements from radar and the inertial unit;
2. radar/EO tracking mode where current estimates of obstacle relative position and velocity are determined also on the basis of EO sensor measurements.

In this paper, the radar-only tracking configuration was considered in order to evaluate the impact of particle filtering techniques first in a single sensor framework. Indeed, given the hierarchical architecture of the considered system, radar-only tracking performance has a significant impact on data fusion operation. In particular, while in TECVOL project the tracking module was based on an EKF-based central-level fusion system for real time applications, this paper discusses the development of a particle filter technique which has been tested in off-line simulations; in fact, innovative techniques are expected to provide more accurate estimates of obstacle dynamics than EKF in case of nonlinear systems, thus reducing the delay in collision detection.

## 4. Developed Software Description

In general, the tracking software allows effective fusion of information provided by different sensors, such as radar and inertial unit; moreover it performs the gating/association functions in order to associate at the same intruder measurements gathered in different scans and to eliminate false alarms and clutter returns. In fact, the inputs to the algorithm are sensor measurements, which represent in general object of interest, false alarm, and clutter. In this case, most of the clutter return will likely come from ground echoes. Track/measurement association is carried out using ellipsoidal gating and a centralized statistic [31] defined as

(3) $$\begin{array}{c} \xi ={\left[\overset{-}{y}-\hat{y}\right]}^{\prime }{\left[R+HPH^{\prime }\right]}^{-1}\left[\overset{-}{y}-\hat{y}\right], \end{array}$$

where $\overset{-}{y}$ is the measurement vector at the time of detection, $\hat{y}$ is the predicted measurement vector at the time of detection, *P* is the predicted covariance matrix, *H* is the measurement transition matrix, and *R* is the measurement covariance matrix. This statistics is a normalized distance between measurement and prediction, which takes into account all the relevant uncertainties. Assuming a statistical distribution for *ξ*, it is then possible to define a threshold based on the probability of a correct measurement falling inside the gate. As an example, in common radar applications *ξ* is assumed to be distributed as a chi-square variable with a number of degrees of freedom equal to the dimension of the measurement vector.

After the association phase, based on a common nearest neighbour approach, since a dense multitarget environment is unlikely to be found in the considered civil collision avoidance scenario, the track status must be properly handled. In particular, tracks are divided in three categories: one-plot (single observation not associated with any existing track), tentative (at least two observations, but confirmation logic is still required), and firm (confirmed track). The transition tentative firm can be based on different techniques. Tracks are deleted if they are not updated within a reasonable interval.

The filtering and prediction phase allows combining track prediction and sensor measurements and produces new track predictions. The tracking system is based on a particle filter with nonlinear dynamic model and linear measurement equation. The state vector is comprised of 6 components that are the obstacle positions in terms of range, azimuth, and elevation in north-east-down (NED) reference frame and their first time derivatives. Besides, the PF tracker provides also an estimate of the Distance at Closest Point of Approach. The obstacle dynamic model is based on a nearly constant velocity model better described in the following section. Important points are related to the sensor latency and the proper inclusion of aircraft navigation measurements. Thus, proper data registration techniques have been considered as a prerequisite for the developed algorithm [32]. Regarding time registration, the radar sends its measurements at the end of each pass with maximum latency of the order of 1 s. Since the tracker works at 10 Hz, in the developed system a measurement is assigned a time stamp, which is the nearest time on the tracker 0.1 s scale. Since the radar delay is typically larger than the 0.1 s scale, it is likely that a measurement relative to time *t*_1_ arrives when the state has already been propagated to a later time *t*_*n*_. In this case, the state and covariance must be updated at time *t*_1_. Also, the gating and association processes have to be performed with values of state and covariance at time *t*_1_. Then, the problem is how to produce updated parameters from measurement at time *t*_1_.

In order to perform gating, association, and track updating, the solution is to keep in memory a sliding window where navigation and track data are stored. The considered time window dimensions correspond to the largest possible radar data latency. Given that state and covariance at time *t*_1_ have to be known and stored, in the developed PF tracker, the key idea is to consider all the variables at time *t*_1_, to perform the filtering step taking into account the radar measurement at that time, and then update the state (and covariance) until time *t*_*n*_ by means of a Monte Carlo approach.

### 4.1. Particle Filter Algorithm

Particle filters are Bayesian estimators that resolve the state estimation problems by determining the probability density function (PDF) of an unknown random vector using a weighted sum of delta functions. These filters have less limitations than the Kalman filter. Indeed, they can exploit nonlinear process and measurement models and they can be used with any form of system noise statistical distribution.

The implemented particle filter is based on the sampling importance resampling algorithm [33] constituted by three main steps: generation of particles, calculation of the weights associated with the particles, and resampling procedure. Then, the state space is propagated through a nonlinear dynamic equation. A brief overview of the PF phases is reported.

Let us consider a set of points and associated weights in the target state space {*w*_*k*−1_^(*i*)^, *x*_*k*−1_^(*i*)^} for *i* = 1,…, *N*_*s*_, which represent samples from the updated target state PDF at time *k* − 1. The latter can be represented by a weighted sum of delta functions as

(4) $$\begin{array}{c} p\left(x_{k-1}\;|\;Z^{k-1}\right)\approx \overset{N}{\underset{i=1}{\sum }}w_{k-1}^{\left(i\right)}\delta \left(x_{k-1}-x_{k-1}^{\left(i\right)}\right), \end{array}$$

where *w*_*k*−1_^(*i*)^ is the weight associated with the *i*th particle and ∑_*i*=1_^*N*^ *w*_*k*−1_^(*i*)^ = 1.

In the developed algorithm, the target state space (i.e., the points and related weights) is initialized generating the particles from a multivariate normal distribution with mean equal to the first radar measurements and specified standard deviation values. In particular, the uncertainties have been estimated on the basis of obstacle dynamic behavior and radar accuracy (*σ*_*r*_ = 9 m, *σ*_*ϑ*_ = 2°, *σ*_*ϕ*_ = 2°). Choosing a large initial uncertainty on obstacle position allows letting a good number of particles representing the state in important or high likelihood regions. Consequently, radar measurements have non-negligible importance in the track initialization phase. At the first step, all the particles are generated with the same weight set equal to* 1/N*. After that, the set of points representing *p*(*x*_*k*−1_∣*Z* ^*k*−1^) have to be transformed into a set of points {*w*_*k*_^(*i*)^, *x*_*k*_^(*i*)^} representing *p*(*x*_*k*_∣*Z* ^*k*^). This is done by PF in two steps: prediction and filtering.

The prediction step exploits the system model to predict the state distribution function from one measurement time to another one. It transforms the updated density at time *k* − 1 into a set of points representing the predicted density *p*(*x*_*k*_∣*Z* ^*k*−1^) by substituting each sample *x*_*k*−1_^(*i*)^ in the dynamic equation. Since the state is subject to a process noise modeled as an independent and identically distributed process noise sequence, the prediction step translates, deforms, and spreads the state distribution.

The filtering step utilizes a measurement at time *k* to update the set of particles representing the propagated density *p*(*x*_*k*_∣*Z* ^*k*−1^) to a set of particles {*w*_*k*_^(*i*)^, *x*_*k*_^(*i*)^} ~ *p*(*x*_*k*_∣*Z* ^*k*^) with updated weights. The optimal choice is to sample from the updated density *p*(*x*_*k*_∣*Z* ^*k*^), directly; since this is impossible because it is unknown, the solution is to sample from the propagated density (as in this specific analysis). Then, given a measurement *z*_*k*_ and the set of propagated points {*w*_*k*−1_^(*i*)^, *x*_*k*_^(*i*)^}, the updated set of particles is given by

(5) $$\begin{array}{c} w_{k}^{\left(i\right)}=\frac{w_{k-1}^{\left(i\right)}p\left(z_{k}\;|\;x_{k}^{\left(i\right)}\right)}{\sum_{j=1}^{N}w_{k-1}^{\left(j\right)}p\left(z_{k}\;|\;x_{k}^{\left(j\right)}\right)}, \end{array}$$

where *p*(*z*_*k*_∣*x*_*k*_^(*i*)^) is the measurement likelihood; in this application it has been defined as

(6) $$\begin{array}{c} p\left(z_{k}\;|\;x_{k}\right)=\frac{1}{\sqrt{2\pi {||R||}^{2}}}\exp\left(-\frac{{\left(z_{k}-{\hat{z}}_{k}\right)}^{\prime }R^{-1}\left(z_{k}-{\hat{z}}_{k}\right)}{2}\right), \end{array}$$

where ${\hat{z}}_{k}$ is the predicted measurement at the time of detection and *R* is the measurement covariance matrix.

In the software the resampling procedure is based on a systematic resampling scheme [33] which is performed at each time step and not only when the number of particles with non-negligible weight falls below a threshold value. The resampling phase enables a great quantity of particles to survive during the propagation of the state space, thus avoiding the degeneracy phenomenon.

The software outputs based on range, azimuth, elevation and their first time derivatives are then calculated on the basis of the state estimates weighted on all particles.

**Figure 4:**
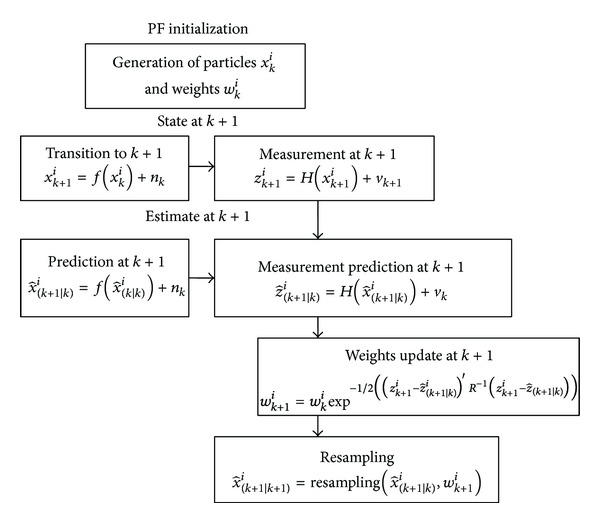
Particle filtering algorithm.

In Figure 4 a particle filtering scheme is reported in order to better clarify how the developed software has been conceived. The scheme exploits the main steps of the algorithm: initialization, prediction, filtering, and resampling. In particular, the filtering phase is based on weights update obtained considering measurement prediction, sensor outputs, and measurement covariance matrix *R*.

### 4.2. Obstacle Tracking Dynamic Model

The selection of a dynamic model is a nontrivial problem for airborne obstacle tracking systems, since an accurate estimate of the obstacle position is considered fundamental for the evaluation of the Distance at Closest Point of Approach. In the developed software, a nearly constant velocity model in spherical coordinates has been implemented to represent the target relative trajectory [34, 35]. The target state is propagated through a nonlinear dynamic equation since the state variables are in spherical coordinates and thus closer to sensor measurements outputs.

The state vector is comprised of obstacle position in terms of range, azimuth, and elevation in north-east-down reference frame and their first time derivatives. Hereinafter, the obstacle dynamic model is reported for the sake of clarity.

The target state vector in spherical coordinates is defined as

(7) $$\begin{array}{c} x=\left[r,\dot{r},\vartheta ,\dot{\vartheta },\phi ,\dot{\phi }\right], \end{array}$$

where *r* is the range, *ϑ* is the azimuth, *ϕ* is the elevation angle, and $\dot{r}$, $\dot{\vartheta }$, $\dot{\phi }$ are their first time derivatives.

In order to evaluate the velocity components, let us consider

(8) $$\begin{array}{c} v=\left[\begin{array}{c} v_{r}\\ v_{\vartheta }\\ v_{\phi } \end{array}\right]=\left[\begin{array}{c} \dot{r}\\ r\dot{\vartheta }\cos\phi \\ r\dot{\phi } \end{array}\right], \end{array}$$

where the velocity vector has been defined as *v* = [*v*_*r*_, *v*_*ϑ*_, *v*_*ϕ*_] for the sake of simplicity.

For a spherical constant velocity dynamic model representing the object motion, it is assumed that ${\dot{v}}_{r}={\dot{v}}_{\vartheta }={\dot{v}}_{\phi }=0$. Taking the time derivative of each component in (8) yields

(9) $$\begin{array}{c} {\dot{v}}_{r}=\ddot{r}=0,\\ {\dot{v}}_{\vartheta }=\dot{r}\dot{\vartheta }\;\cos\;\varphi +r\ddot{\vartheta }\;\cos\;\varphi -r\dot{\vartheta }\dot{\varphi }\;\sin\;\varphi =0,\\ {\dot{v}}_{\phi }=\dot{r}\dot{\varphi }+r\ddot{\varphi }=0. \end{array}$$

Rearranging the above expressions in terms of angular velocities and considering discrete-time domain with sampling time *T*, the components of the dynamic motion equation are given by

(10) $$\begin{array}{c} r_{n}=r_{n-1}+T{\dot{r}}_{n-1},\\ {\dot{r}}_{n}={\dot{r}}_{n-1},\\ \vartheta_{n}=\vartheta_{n-1}+T{\dot{\vartheta }}_{n-1}\left[1+\frac{T}{2r_{n-1}}\left(r_{n-1}{\dot{\phi }}_{n-1}tg\phi_{n-1}-{\dot{r}}_{n-1}\right)\right],\\ {\dot{\vartheta }}_{n}={\dot{\vartheta }}_{n-1}\left[1+T\left({\dot{\phi }}_{n-1}tg\phi_{n-1}-\frac{{\dot{r}}_{n-1}}{r_{n-1}}\right)\right],\\ \phi_{n}=\phi_{n-1}+T{\dot{\phi }}_{n-1}\left[1-\frac{T}{2r_{n-1}}{\dot{r}}_{n-1}\right],\\ {\dot{\phi }}_{n}={\dot{\phi }}_{n-1}\left[1-\frac{T}{2r_{n-1}}{\dot{r}}_{n-1}\right]. \end{array}$$

The nonlinear dynamic motion equation for the spherical state vector can be written as

(11) $$\begin{array}{c} x_{n}=f\left(x_{n-1}\right)+n_{n-1} \end{array}$$

with the zero-mean Gaussian dynamic acceleration noise distribution

(12) $$\begin{array}{c} n_{n}\sim N\left(0,Q_{\rho }\right). \end{array}$$

The components of *f*(*x*_*n*−1_) are given by the nonlinear equation (10) and the process noise matrix is

(13) $$\begin{array}{c} Q_{\rho }=\left[\begin{array}{ccc} q_{r}Q_{1} & 0_{2} & 0_{2}\\ 0_{2} & q_{h}Q_{1} & 0_{2}\\ 0_{2} & 0_{2} & q_{v}Q_{1} \end{array}\right] \end{array}$$

with

(14) $$\begin{array}{c} Q_{1}=\left[\begin{array}{cc} \frac{T^{3}}{3} & \frac{T^{2}}{2}\\ \frac{T^{2}}{2} & T \end{array}\right], \end{array}$$

where *q*_*r*_, *q*_*h*_, and *q*_*v*_ must be set depending on the target maneuvers [34].

Since the model is in spherical coordinates, the measurement equation is linear and it can be expressed as

(15) $$\begin{array}{c} z_{n}=Hx_{n}+w_{n} \end{array}$$

with

(16) $$\begin{array}{c} H=\left[\begin{array}{cccccc} 1 & 0 & 0 & 0 & 0 & 0\\ 0 & 0 & 1 & 0 & 0 & 0\\ 0 & 0 & 0 & 0 & 1 & 0 \end{array}\right] \end{array}$$

and *w*_*n*_ is the observation noise which is considered to be independent zero-mean Gaussian noise defined by

(17) $$\begin{array}{c} w_{n}\sim N\left(0,R_{n}\right), \end{array}$$

where *R*_*n*_ is the measurement covariance matrix, as stated above.

The developed obstacle detection and tracking software provides also an estimate of the Distance at Closest Point of Approach (see (1)). Since the particles are characterized by own positions and velocities and given the nonlinear dependencies between $\bar{r}$, $\bar{V}$, and DCPA, the latter is calculated for each particle. In this way, in order to avoid further approximations due to the evaluation of this distance from position and velocity mean values, DCPA has been calculated as mean value of the different distances evaluated as

(18) $$\begin{array}{c} \text{DCP}{\text{A}}_{n}=\left|\frac{\overset{-}{r}\cdot \bar{V}}{{||\bar{V}||}^{2}}\bar{V}-\bar{r}\right|. \end{array}$$

This is a form of “deep integration” of a collision detection sensor since an estimate of the level of collision threat is determined by means of the same data fusion algorithm. This is an additional advantage of using PF rather than EKF, since the need of linearized models in EKF does not allow estimating DCPA by the filter itself.

## 5. Results

The developed obstacle tracking algorithm has been tested in off-line simulations based on flight data gathered during an intensive test campaign. Flight tests were carried out by exploiting the following configuration of test facilities:

- FLARE aircraft piloted by a human pilot or by the autonomous flight control system;
- a piloted intruder VLA aircraft in the same class of FLARE equipped with GPS antennas and receivers, and an onboard CPU for navigation data storage;
- a ground control station (GCS) for real time flight coordination and test monitoring [36];
- a full-duplex data link between FLARE and GCS, used to send commands to initiate or terminate tests and to receive synthetic filter output and navigation measurements;
- a downlink between intruder and GCS used for flight monitoring.

During the tests two types of maneuvers were executed, such as chasing tests with FLARE pursuing the intruder and quasi-frontal encounters performed to estimate detection and tracking performance in the most interesting scenarios from the application point of view.

In particular, the scenario selected in this paper for performance analysis is a single quasi-frontal encounter between FLARE and the intruder. The algorithm has been tested in standalone radar tracking configuration; that is, estimates of obstacle relative position and velocity are based on measurements from radar (update frequency of 1 Hz) and the own-ship navigation system.

The number of particles has been set equal to 500. This number was selected since increasing it up to one order of magnitude did not produced significant performance increase. The algorithm is initialized taking into account the first radar sensor measurement; the covariance matrix is calculated on the basis of sensor measurements and then it is updated with estimated values of software output.

The algorithm outputs are based on obstacle position and velocity in terms of range, azimuth, and elevation and DCPA in a stabilized north-east-down reference frame with origin in FLARE center of mass. The considered flight segment has duration of about 20 s with an initial range of about 2000 m.

**Figure 5:**
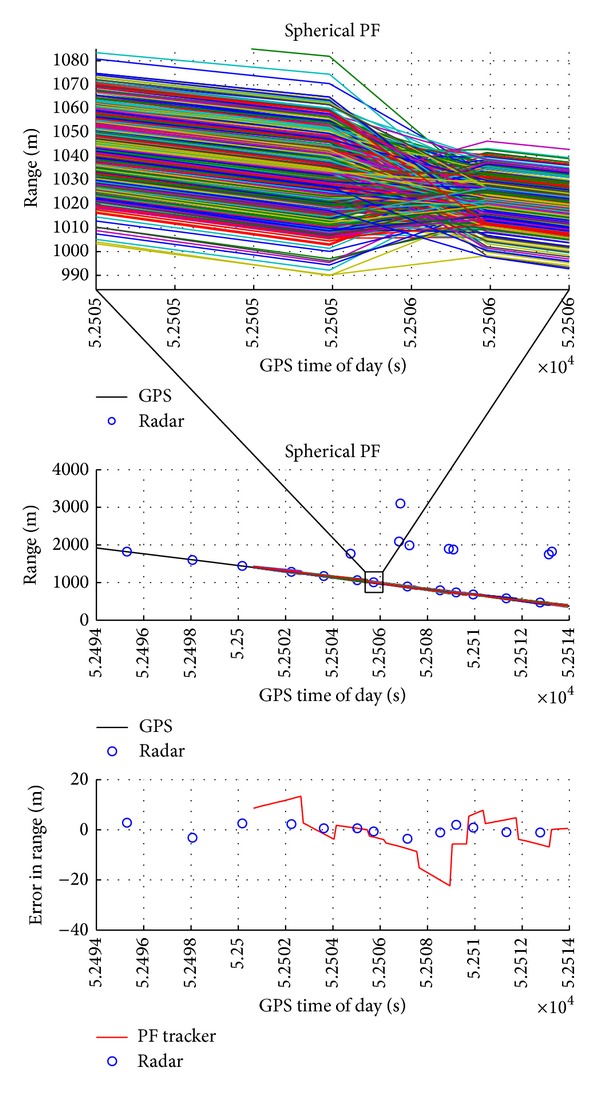
Range as estimated by GPS, by radar, and by radar-only tracker and estimation error as a function of GPS time of day. The upper figure highlights the trajectory of each particle.

In Figure 5, obstacle range as estimated by radar, radar-based tracker, and GPS (reference) together with error estimate with respect to GPS measurements is reported. After a firm track has been generated on the basis of radar measurements, the tracker is able to characterize the obstacle trajectory. The radar-only tracker provides a range estimate with an accuracy of the order of few meters that derives from the good radar range accuracy and the absence of intruder maneuvers. In Figure 5, the upper plot illustrates the obstacle range in a small interval time (of about 1 s) in order to show the particles trajectories (colored lines).

**Figure 6:**
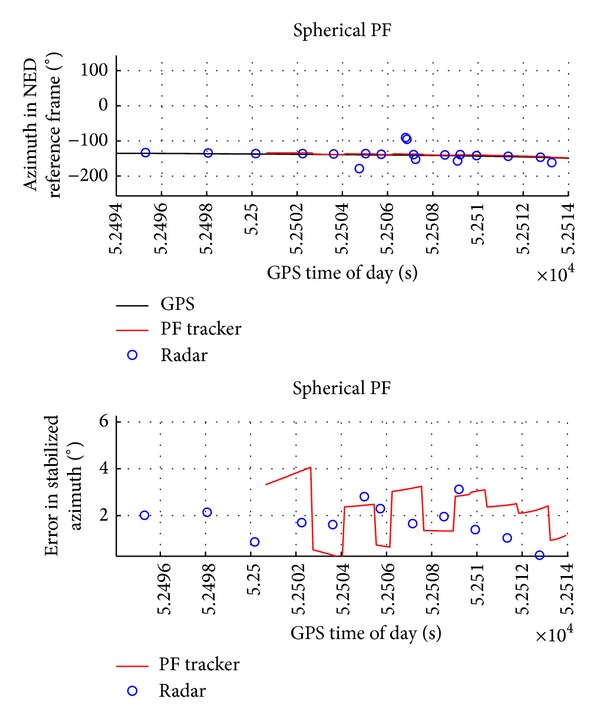
Azimuth in NED reference frame as estimated by GPS, by radar, and by radar-only tracker and estimation error as a function of GPS time of day.

**Figure 7:**
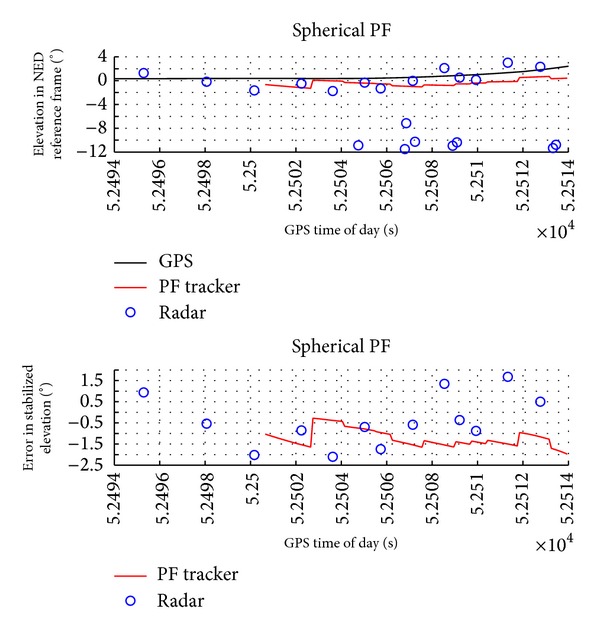
Elevation in NED reference frame as estimated by GPS, by radar, and by radar-only tracker and estimation error as a function of GPS time of day.

Figures 6 and 7 report the obstacle angular dynamics in terms of azimuth and elevation. As expected, the order of magnitude of these errors is comparable to the radar accuracy. In particular, the estimation uncertainty in terms of root mean square is of about 2.5° for azimuth and 1.3° for elevation. Being computed in NED, these RMS errors include attitude measurement error biases. In fact, error biases in the estimation of magnetic heading are the main reason of the larger error in azimuth. It is worth noting that these biases have a little effect on the estimation of angular rates and of Distance at Closest Point of Approach.

**Figure 8:**
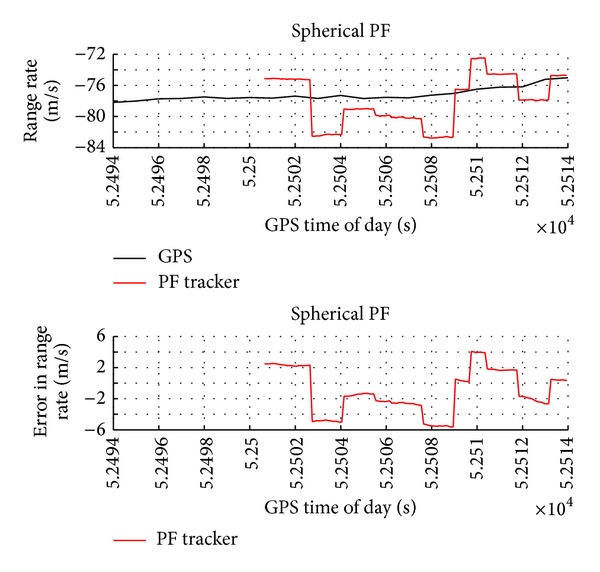
Range rate as estimated by GPS and by radar-only tracker and estimation error as a function of GPS time of day.

**Figure 9:**
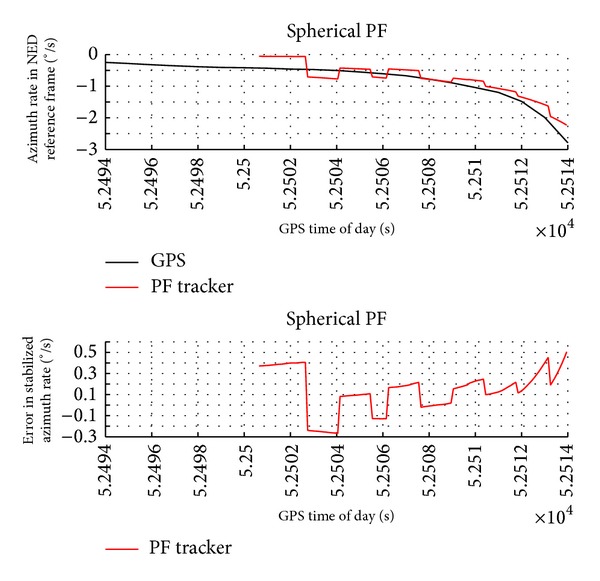
Azimuth rate in NED reference frame as estimated by GPS and by radar-only tracker and estimation error as a function of GPS time of day.

**Figure 10:**
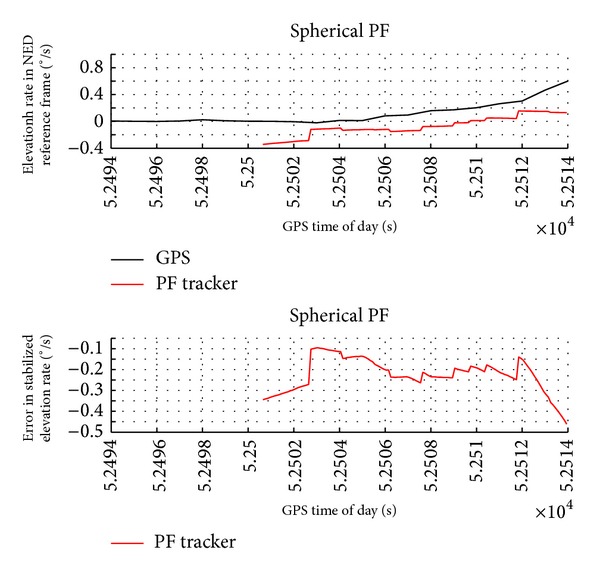
Elevation rate in NED reference frame as estimated by GPS and by radar-only tracker and estimation error as a function of GPS time of day.

The range and angular rates as estimated by GPS (reference) and particle filter tracker are reported in Figures 8, 9, and 10. These variables are very important information sources for collision detection assessment even though they are not directly provided by the radar sensor. The plots show that the tracker estimates are very accurate with a very small value in terms of standard deviation.

Simulation results confirm how the resampling procedure is a key factor for tracking performance, avoiding the degeneracy phenomenon and thus enabling a great quantity of particles to survive to the prediction step.

In order to better understand the particle filter tracking capability, PF and EKF estimation errors are reported in Table 1 in terms of root mean square (RMS) errors computed as

(19) $$\begin{array}{c} \text{RMS}=\sqrt{\frac{1}{N}\overset{N}{\underset{t=1}{\sum }}{\left({\hat{x}}_{t}-x_{t}\right)}^{2},} \end{array}$$

where *N* is the number of measurements, ${\hat{x}}_{t}$ is the generic PF tracker estimate (computed after resampling and representing the mean value over all the particles), and *x*_*t*_ is the reference value computed on the basis of FLARE and intruder GPS measurements.

The results show that PF tracker performance is comparable to EKF in terms of order of magnitude. Of course the introduction of electro-optical data can improve angular accuracy since these sensors have a faster update rate than radar. In particular, while the range rate error is very similar for the two filters, obstacle angular velocities provided by PF algorithm are slightly more accurate than EKF in the considered scenario. These variables are very important information sources for collision detection assessment even though they are not directly provided by the radar.

In fact, in near collision conditions angular rates are the most important variables influencing collision detection. As shown in [7], in these conditions DCPA uncertainty for given range and range rate shows a linear dependence on angular rate errors, while range rate essentially impacts the time-to-go estimate.

**Figure 11:**
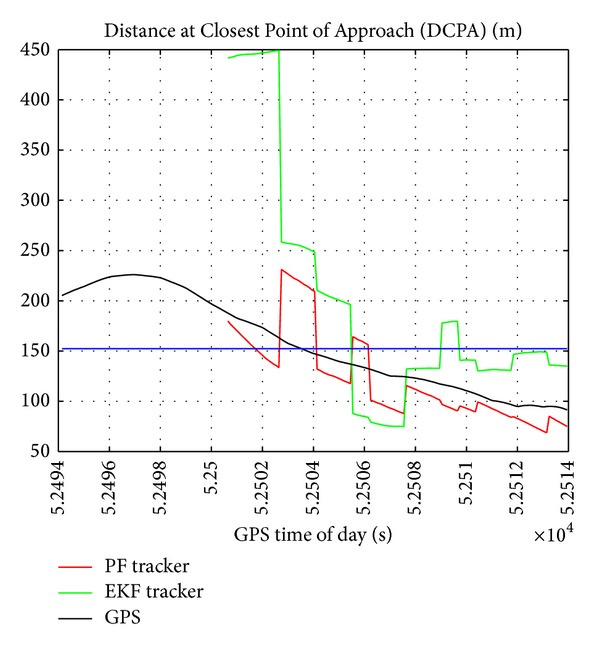
Distance at Closest Point of Approach as estimated by GPS (reference), by radar, and by radar-only tracker as function of GPS time of the day.

In the considered scenario, DCPA as computed on the basis of GPS measurement has been used as a reference for comparing DCPA estimated by EKF tracker and PF tracker.  Figure 11 shows that the PF algorithm is more accurate than EKF for the estimation of the DCPA in the initial phase of firm tracking (at larger range), thus providing an improvement in collision risk assessment and a potential reduction in the delay in declaring a collision threat. Analysis of GPS data demonstrates that the considered encounter represents a real Near Mid-Air Collision (NMAC) scenario, since the reference DCPA keeps a very small value and lies below the bubble distance threshold almost in the whole firm tracking phase.

## 6. Conclusion and Future Works

This paper focused on algorithm and test results from obstacle tracking software based on particle filter and aimed at demonstrating UAS autonomous collision detection capability in terms of reliable estimation of Distance at Closest Point of Approach. The algorithm performance has been initially evaluated in radar-only configuration since the main interest was addressed to the analysis of the potential impact of nonlinear filters on tracking capabilities with respect to assessed EKF first in a single sensor framework.

The software has been tested in off-line simulations based on real data recorded during a flight test campaign, and a quasi-frontal encounter between the own aircraft and the intruder has been presented as test case. The results pointed out that the developed algorithm is able to detect and track the obstacle trajectory with accuracy comparable to the EKF. In particular, some advantages were highlighted in the direct determination of Distance at Closest Point of Approach, with a potential reduction of the delay for collision detection. This result permits planning additional future investigation activities, such as

- performing the comparison on a larger experimental data set, thus increasing the statistical significance;
- developing a multisensor-based analysis to confirm the results obtained by the developed PF tracker when additional data sources are available, such as electro-optical sensors; indeed, they can provide higher accuracy in angular measurements and better data rate than radar sensors, with a great impact on DCPA performance.

## Figures and Tables

**Table 1:** PF performance in the selected scenario in terms of RMS error.

RMS	Particle filter	EKF
Range (m)	8.4	3.7
Range rate (m/°)	3.0	2.3
Azimuth (°)	2.5	1.7
Azimuth rate (°/s)	0.2	0.4
Elevation (°)	1.3	2.1
Elevation rate (°/s)	0.2	0.8
